# How Useful Is the SSN–Nipple Distance? An Analytical Questionnaire Survey on Anthropometric Measurements for the Aesthetically Ideal Positioning of the Nipple–Areolar Complex

**DOI:** 10.3390/jcm12072494

**Published:** 2023-03-25

**Authors:** Rafael Loucas, Marios Loucas, Sebastian Leitsch, Julius Michael Mayer, Andrea Alberti, Omar Haroon, Marlon Petrus, Konstantin Christoph Koban, Thomas Holzbach

**Affiliations:** 1 Department of Hand and Plastic Surgery, Thurgau Hospital Group, 8500 Frauenfeld, Switzerland; 2 Division of Plastic, Aesthetic and Reconstructive Surgery, Medical University of Graz, 8010 Graz, Austria; 3 Department of Plastic and Hand Surgery, Inselspital, University Hospital Bern, 3010 Bern, Switzerland; 4 Department of Plastic, Reconstructive and Aesthetic Surgery, Ospedale Regionale di Lugano, Ente Ospedaliero Cantonale (EOC), 6900 Lugano, Switzerland; 5 Division of Hand, Plastic and Aesthetic Surgery, University Hospital LMU Munich, 80339 Munich, Germany

**Keywords:** breast, symmetry, nipple, aesthetic breast, mid-clavicle-to-nipple distance, suprasternal-notch-to-nipple distance, survey, nipple–areolar complex

## Abstract

Several studies have attempted to identify the optimal anthropometric measurement for the aesthetically ideal positioning of the nipple–areolar complex. However, no standardised solutions and measurements for planning surgical procedures have been reached. The aim of this study is to identify the optimal anthropometric measurement between the suprasternal notch (SSN)–nipple distance and mid-clavicle (MC)–nipple distance for the aesthetic position of the nipple–areola complex (NAC) on the breast. A detailed online survey was sent to 300 board-certified plastic surgeons and residents of plastic surgery departments of hospitals in German, Austrian, and Swiss. A similar survey was also provided to 100 patients who had planned or had already undergone breast surgery. All participants were asked to rank the attractiveness of a series of women’s breasts in images with different NAC position measurements. The images showed breasts from two different measurements and distances: all the breasts had equal dimensions and proportions and the same areola size. Complete datasets were obtained from 203 of the 300 board-certified plastic surgeons and residents of plastic surgery departments in German-speaking countries (recall 68%) and from 100 patients. The majority of doctors and patients find a symmetrical breast with a mirrored position of the nipple–areola complex more attractive than a non-symmetrical breast. In cases with minor measurement differences, such as 0.5 cm (SSN vs. MC), there is no relevant difference in the breast symmetry. However, at larger distances, the MC-to-nipple distance is superior for achieving aesthetically appealing symmetry compared with the SSN-to-nipple distance. Using the MC-to-nipple distance seems to be superior for correct nipple positioning than the SSN-to-nipple distance and is a valuable preoperative measurement option for breast symmetry with correct nipple height. Further studies on this topic involving a more general population should be conducted to confirm the improvements in perception with the preoperative measurements using the anatomical landmarks.

## 1. Introduction

Precise preoperative markings prove indispensable for state-of-the-art plastic and reconstructive surgery [1,2,3,4], particularly in breast surgery, which mainly relies on the conventional anthropometric measurement of distances in correlation to anatomical landmarks [3,5,6,7]. Regardless of the nature of the procedure, whether reconstructive or cosmetic, the correct nipple position is a key factor in the perception of breast symmetry and significantly affects the overall appearance of a woman’s breast [8,9,10]. Recent studies have attempted to identify the ideal nipple–areola complex (NAC) localisation and provide an objective description of the aesthetically ideal breast. However, they either focus on isolated parameters or suffer from a lack of reliability or measurable results [8,11,12,13,14]. Consequently, the evaluation of the optimal anthropometric measurement for the aesthetically ideal position of the NAC is still poorly defined [8,11,12,13,14]. A variety of measurements are utilised to determine the proper nipple–areola position. The most commonly used measurements in determining the correct nipple height are the suprasternal notch (SSN)-to-nipple distance and the mid-clavicle (MC)-to-nipple distance [3,5,6,15,16]. Although both distances lack precision, these are the most used anatomical landmarks [3,5,6,15,16].

The SSN-to-nipple distance does not account for a symmetrical nipple height when the NAC is not symmetrically centred in both breasts or if there is a difference in breast width [17]. Here, the MC-to-nipple distance appears to be a superior measure but is still prone to inaccuracies when there is a difference in clavicle length or clavicle height. Thus, the aim of this study is to compare the two most prevalent measurements for NAC positioning, namely, the SSN-to-nipple distance and the MC-to-nipple distance, for a more aesthetically pleasing outcome of the position of the NAC on the breast. Furthermore, we evaluate whether we see differences between patients’ perception and plastic surgeons’ professional opinion. 

## 2. Materials and Methods

We designed a cross-sectional questionnaire study for hospitals in German-speaking countries and our patients. A detailed online survey was sent by email to 300 board-certified plastic surgeons and residents of plastic surgery departments of hospitals in Germany, Austria, and Switzerland. A similar survey was also provided to 100 patients who plan to undergo or have undergone breast surgery. All participants were asked to rank the attractiveness of a series of women’s breasts in images with different NAC position measurements. The images showed computer-simulated breast models with two different measurements and distances: all the breasts had equal dimensions and proportions with the same areola size. Furthermore, the questionnaire was designed to be anonymous to acquire a higher response rate and more thoughtful answers, and, consequently, unbiased data. The survey also queried the current range of subspecialties offered in hospitals in German-speaking countries.

### 2.1. Design of the Questionnaire 

The online survey was designed to determine the optimal anthropometric measurement between the SSN–nipple distance and the MC–nipple distance for the best aesthetic position of the NAC on the female breast using the responses to the questionnaire. The questionnaire was entered into the UmfrageOnline (enuvo GmbH, Pfaeffikon, Switzerland) system and was made available in four languages: English, German, Italian, and French. 

In the first part of the survey, the participants were asked to provide their age, work experience, and their plastic-surgical spectrum (questions 1, 2, and 3). The second part presented a series of frontal computer-simulated breast models with different NAC position measurements. The images showed breasts with two different measurements and distances, with 16 frontal-view images of both the breasts shown. All the breasts had equal dimensions and proportions with the same areola size. The simulations were arranged in pairs with one side altering only the SSN-to-nipple distance and the other altering only the MC-to-nipple distance. No auxiliary or reference lines were displayed in these pictures, and no subsidiary information was provided. The alterations of SSN-to-nipple or MC-to-nipple distances were randomly distributed to the right side or left side of the paired pictures. All the participants were asked to pick the side that appeared more attractive to them. 

### 2.2. Positioning of the NAC

**Figure 1:**
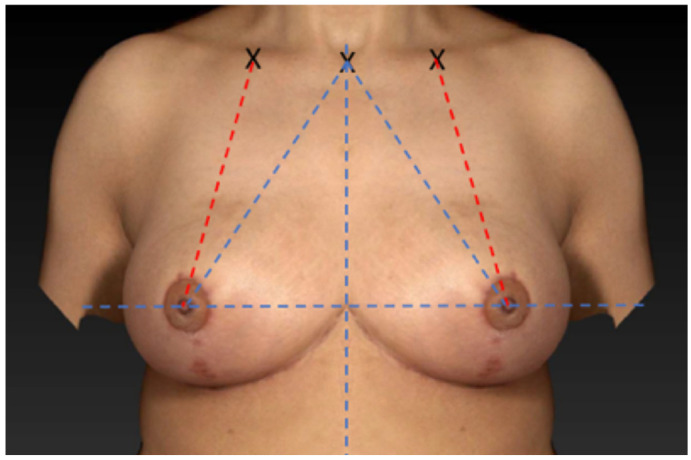
Symmetrical mirrored computer-simulated breast model.

To place the NAC in different positions according to the measurement method, all computer-simulated breast images showed the same mirrored image, with one side modified according to the measurement method used (Figure 1). The first two pairs of images showed a 100% symmetrical mirrored image and a non-symmetrical image. Three paired computer-simulated breast models showed the same mirrored image where one side was modified to vary 5, 10, or 15 mm in the SSN-to-nipple distance while maintaining the MC-to-nipple distance as symmetrical. The next set of three paired computer-simulated breast images was altered to vary 5, 10, or 15 mm in the MC-to-nipple distance while maintaining the SSN-to-nipple distance as symmetrical. The different NAC positions were reached using a coordinate system (*x* and *y* axes), while the NAC was displaced horizontally and vertically. Adobe Photoshop^®^ (Adobe Inc., San Jose, CA, USA) was used for mirroring the images and the different positioning of the NAC.

### 2.3. Statistical Analysis

The results were collected and analysed descriptively using graphs and statistics. The participant’s information and the differences between the computer-simulated breast models were compared using an independent samples t-test for continuous variables and chi-square analyses for categorical variables to detect differences between the groups. A difference of *p* < 0.05 was considered to be statistically significant. All statistical analyses were processed with the IBM SPSS^®^ statistics software (version 28.0; Chicago, IL, USA).

## 3. Results

### Questionnaire Results

**Figure 2:**
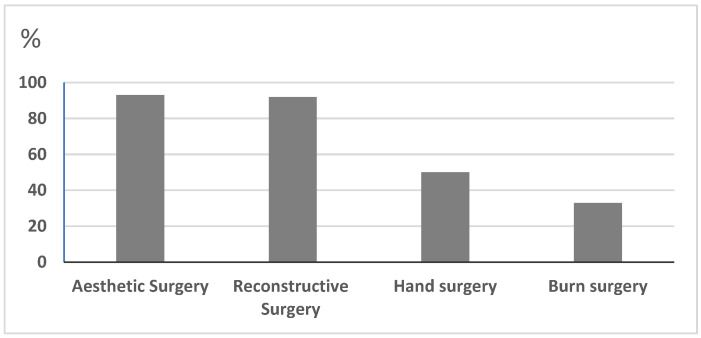
The professional spectrum of the participants in the departments in German-speaking countries.

Complete datasets were obtained from 203 of the 300 board-certified plastic surgeons and residents of plastic surgery departments in German-speaking countries (recall 68%) and from 100 patients. All the patients were women, and the average age was 44.3 ± 11.1 years. In total, 84% of the doctors were specialists, and 16% were residents in plastic, reconstructive, and aesthetic surgery. Moreover, 28% of the doctors were women, and 72% were men. Nearly all doctors (93%) performed aesthetic surgery and reconstructive surgery (92%), while only 50% performed hand surgery. About one-third (33%) of the participants in German-speaking countries performed burn surgery (Figure 2). All the patients (100%) had already undergone breast surgery. 

**Figure 3:**
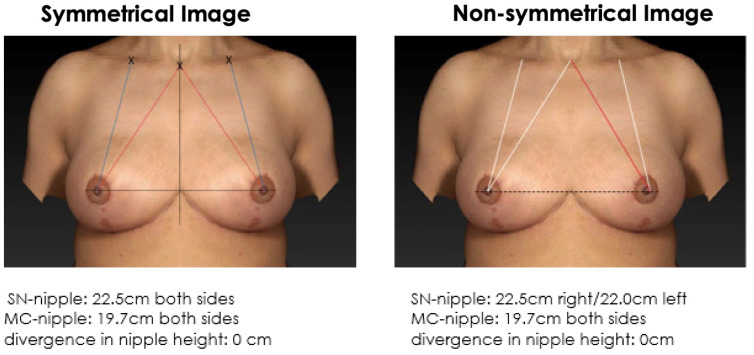
Symmetrical mirrored computer-simulated breast model and a non-symmetrical model (with a modified SSN-to-nipple distance). In total, 89% of the plastic surgeons and 81% of the patients find a symmetrical breast with mirrored position of the NAC more attractive than a non-symmetrical breast.

**Figure 4:**
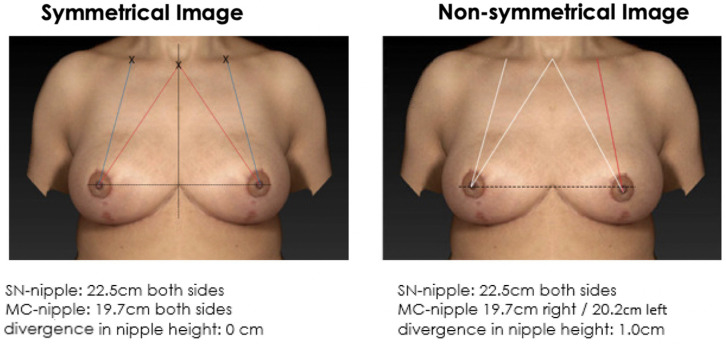
Symmetrical mirrored computer-simulated breast model and a non-symmetrical model (with a modified MC-to-nipple distance). In total, 92% of the plastic surgeons and 89% of the patients find a symmetrical breast with mirrored position of the NAC more attractive than a non-symmetrical breast.

**Figure 5:**
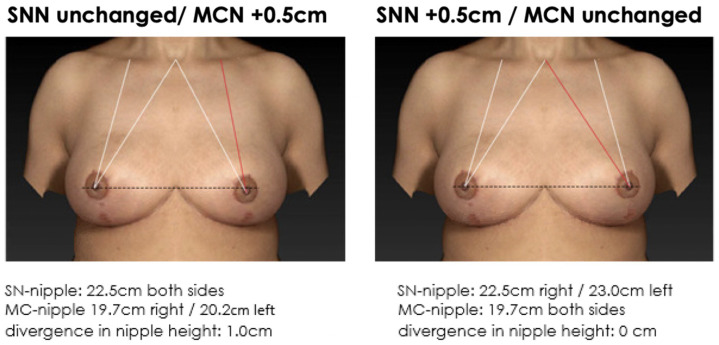
Non-symmetrical mirrored computer-simulated breast models with a modified MC-to-nipple distance and an SSN-to-nipple distance of +0.5 cm. In total, 56% of the plastic surgeons and 60% of the patients find the image with unaltered MC-to-nipple distance more attractive than the image with the unaltered SSN-to-nipple distance.

**Figure 6:**
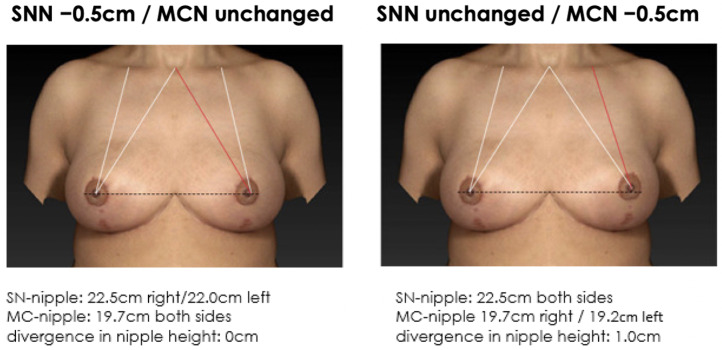
Non-symmetrical mirrored computer-simulated breast models with a modified MC-to-nipple distance and an SSN-to-nipple distance of −0.5 cm. In total, 47% of the plastic surgeons and 59% of the patients find the image with the unaltered MC-to-nipple distance more attractive than the image with the unaltered SSN-to-nipple distance.

**Figure 7:**
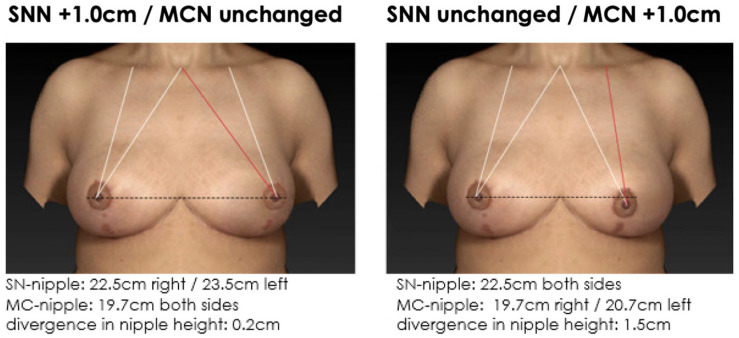
Non-symmetrical mirrored computer-simulated breast models with a modified MC-to-nipple distance and an SSN-to-nipple distance of +1.0 cm. In total, 87% of the plastic surgeons and 79% of the patients find the image with the unaltered MC-to-nipple distance more attractive than the image with the unaltered SSN-to-nipple distance.

**Figure 8:**
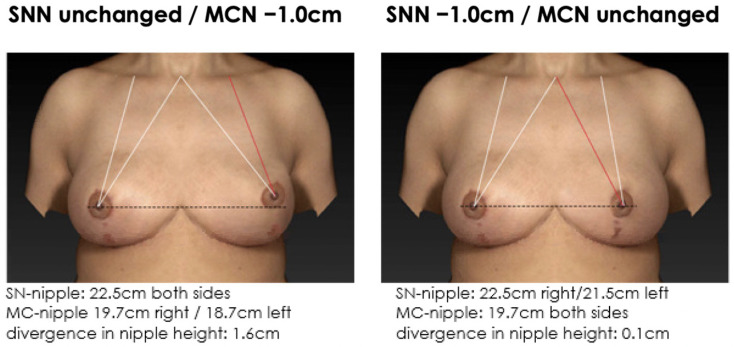
Non-symmetrical mirrored computer-simulated breast models with a modified MC-to-nipple distance and an SSN-to-nipple distance of −1.0 cm. In total, 98% of the plastic surgeons and 99% of the patients find the image with the unaltered MC-to-nipple distance more attractive than the image with the unaltered SSN-to-nipple distance.

**Figure 9:**
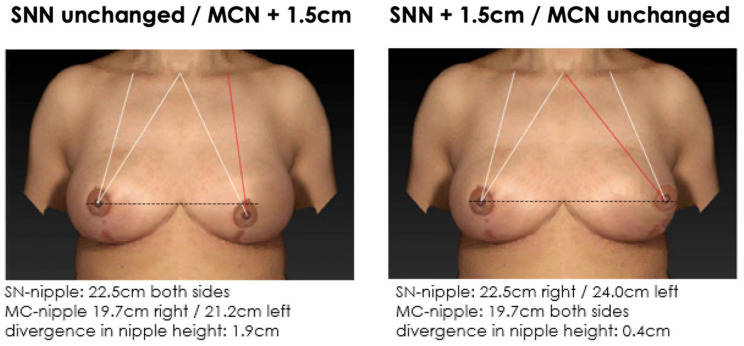
Non-symmetrical mirrored computer-simulated breast models with a modified MC-to-nipple distance and an SSN-to-nipple distance of +1.5 cm. In total, 78% of the plastic surgeons and 69% of the patients find the image with the unaltered MC-to-nipple distance more attractive than the image with the unaltered SSN-to-nipple distance.

**Figure 10:**
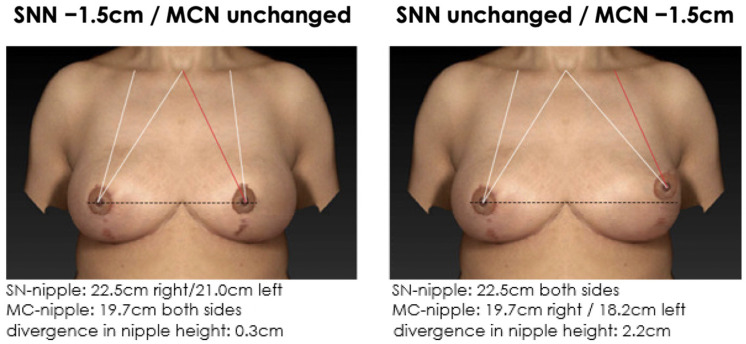
Non-symmetrical mirrored computer-simulated breast models with a modified MC-to-nipple distance and an SSN-to-nipple distance of −1.5 cm. In total, 94% of the plastic surgeons and 96% of the patients find the image with the unaltered MC-to-nipple distance more attractive than the image with the unaltered SSN-to-nipple distance.

The majority of doctors and patients find a symmetrical breast with mirrored position of the NAC more attractive than a non-symmetrical breast (Figure 3 and Figure 4). In cases with minor measurement differences such as 5 mm (SSN vs. MC), no relevant difference is observed in the breast symmetry and respondent’s preference (Figure 5 and Figure 6). However, the MC-to-nipple distance is superior for generating an aesthetically appealing symmetry of the breast at variations between 10 mm and 15 mm than the SSN-to-nipple distance (*p* = 0.03 and *p* < 0.001) (Figure 7, Figure 8, Figure 9 and Figure 10). Different female and male respondents showed no difference in nipple position preferences (*p* = 0.67). Moreover, no relevant difference between doctor and patient respondents was observed (Table 1).

## 4. Discussion

This study shows that the MC-to-nipple distance seems to be superior to the SSN-to-nipple distance for creating aesthetically attractive results and providing a valuable preoperative measurement option for breast symmetry with correct nipple height.

Using anatomical landmarks for correct positioning of the NAC is still under debate. It represents an intellectual and surgical challenge even for experienced plastic surgeons. Different measurement and evaluation options have been proposed for the aesthetically ideal position of the NAC. Lewin et al. attempted to define a template of the aesthetically ideal position of the NAC. They reported that the most preferred NAC placement by both sexes had a ratio of 40:60 *x* and 50:50 *y*, which means that it was best situated in the middle of the breast gland vertically and slightly lateral to the midpoint horizontally. However, they did not use specific anatomical landmarks for positioning the NAC. Therefore, preferences investigated in their study may not coincide with anthropometric measurements and may instead depend, for instance, on images created by mass media [8].

Moreover, Mallucci and Branford attempted to define a template for the ideal female breast [9,10]. They reported that the 45:55 ratio has universal appeal in defining the ideal breast. The authors defined four key features (upper pole–lower pole ratio, nipple angulation, upper-pole slope, and lower-pole convexity) in order to define the aesthetically perfect female breast, with the upper-to-lower pole at a 45:55 ratio, the angulation of the nipple upwards at a mean angle of 20° from the nipple meridian, the upper-pole slope linear or slightly concave, and the lower pole convex. However, the authors assumed that the NAC position was always aligned with the level of maximum breast projection, with the nipple at the upper–lower pole boundary (nipple meridian) and upward pointing (mean angle of 20°) [9,10].

In contrast to all previous studies, a study from 2021 came to the conclusion that based on an online census survey conducted in the United States, the preferred ideal female breast has an upper-pole-to-lower-pole ratio of 55:45 and an areola size of 30 mm [12]. The authors used 3D-generated female models, but they did not investigate the correct position of the NAC. Only parameters such as breast pole ratio, areola size, and breast direction and projection were analysed [12].

Hsia and Thompson also raised the issue that the patient’s preference for an aesthetically ideal breast may differ from what the surgeon considers to be the most aesthetic [18]. They examined the upper-pole slope and reported that although patients often desired a convex upper-pole slope, surgeons preferred a straight or a concave upper-pole slope. Consequently, we were interested in seeking both opinions (surgeons’ and patients’) on the aesthetically ideal positioning of the NAC. Interestingly, in our analysis, both surgeons and patients preferred the breast images in which the difference in the MC-to-nipple distance on both sides was smaller than those images with a slight difference in the SSN-to-nipple distance.

To our knowledge, this is the first prospective cross-sectional questionnaire study seeking opinions from plastic surgeons and it has the largest number of respondents to date. However, we are also aware of some limitations of this investigation. Although the patient data were prospectively enrolled from our institutional database, this study does not fulfil all criteria of a cohort study design. Even after including surgeons and patients, this remains a nonrandomised study with the inherent limitation of such study design and a selection bias (participants were selected based on their status: plastic surgeons or patients seeking or having undergone breast surgery). Furthermore, we decided to include different images with different NAC positions without considering breast shape, volume, or size. When changing the SSN-to-nipple difference but leaving the MC-to-nipple distance unchanged, the NAC leaves both the centre of the breast and the horizontal plane and swings either towards the midline or the side of the breast. The distance from the NAC to the inframammary fold changes as well, while the dimensions of the breast remain identical on both sides.

However, we used simulated images with equal dimensions, equal proportions and the same areola size, while having different NAC positions, with two comparable measurements. This allowed us to objectively ascertain the preferences of the respondents, solely based on the NAC position (which was the only varying factor), independently of the size and proportions of the breasts. 

As a further limitation, we are aware that both anatomical landmarks (the MC-to-nipple distance and the SSN-to-nipple distance) lack precision. The SSN-to-nipple distance depends strongly on the breast width. Considering that the width of the breast indicates the optimal nipple position in the centre of the breast [8], different breast widths could affect the SSN-to-nipple distance, therefore providing a non-symmetrical height of the new nipple position. The same applies to the MC-to-nipple distance. Different clavicle heights or asymmetrical lengths will affect the MC-to-nipple distance, therefore affecting the symmetry of the new nipple position. Additionally, patients undergoing breast surgery do not tape-measure distances but mainly evaluate the correct nipple position depending on their reflection in the mirror [1,17], consequently focusing on the identical height of the NAC of both breasts.

Changes in the MC-to-nipple distance resulted in greater asymmetrical results in nipple height symmetry than those in the SSN-to-nipple distance. Therefore, the MC-to-nipple distance appears to be more relevant for correct nipple positioning than the SSN-to-nipple distance.

## 5. Conclusions

Using the MC-to-nipple distance seems to be superior for correct nipple positioning compared to using the SSN-to-nipple distance and is a valuable preoperative measurement option for achieving breast symmetry with correct nipple height. Further studies on this topic involving a more general population should be conducted to confirm the improvements in perception with the preoperative measurements using the anatomical landmarks.

## Figures and Tables

**Table 1:** Distinguished data between the doctor and patient respondents.

	Doctors; *n* = 203	Patients; *n* = 100	*p* Value	Test
Symmetrical/non-symmetrical image (with modified SSN)	180	81	0.744	Fisher
Symmetrical/non-symmetrical image (with modified SSN)	186	89	0.722	Fisher
SNN unchanged/MCN +0.5 cm	89	40	0.834	Fisher
SNN +0.5 cm/MCN unchanged	114	60	0.911	Fisher
SNN −0.5 cm/MCN unchanged	95	59	0.961	Fisher
SNN unchanged/MCN −0.5 cm	108	41	0.864	Fisher
SNN +1.0 cm/MCN unchanged	177	79	0.899	Fisher
SNN unchanged/MCN +1.0 cm	26	21	0.872	Fisher
SNN unchanged/MCN −1.0 cm	6	9	0.754	Fisher
SNN −1.0 cm/MCN unchanged	198	99	0.983	Fisher
SNN unchanged/MCN + 1.5 cm	45	31	0.843	Fisher
SNN +1.5 cm/MCN unchanged	158	69	0.826	Fisher
SNN −1.5 cm/MCN unchanged	191	96	0.963	Fisher
SNN unchanged/MCN −1.5 cm	12	4	0.989	Fisher

## Data Availability

Supporting data are available from the authors upon request.
